# Identification of genetic elements required for *Listeria monocytogenes* growth under limited nutrient conditions and virulence by a screening of transposon insertion library

**DOI:** 10.3389/fmicb.2022.1007657

**Published:** 2022-10-13

**Authors:** Lakshmi Narayanan, Ozan Ozdemir, Navatha Alugubelly, Reshma Ramachandran, Michelle Banes, Mark Lawrence, Hossam Abdelhamed

**Affiliations:** ^1^Department of Comparative Biomedical Sciences, College of Veterinary Medicine, Mississippi State University, Mississippi State, MS, United States; ^2^Department of Clinical Sciences, College of Veterinary Medicine, Mississippi State University, Mississippi State, MS, United States; ^3^Department of Poultry Science, Mississippi State University, Mississippi State, MS, United States

**Keywords:** *Listeria monocytogenes*, transposon-insertion, virulence, auxotroph, fitness

## Abstract

*Listeria monocytogenes*, the causative agent of listeriosis, displays a lifestyle ranging from saprophytes in the soil to pathogenic as a facultative intracellular parasite in host cells. In the current study, a random transposon (Tn) insertion library was constructed in *L. monocytogenes* strain F2365 and screened to identify genes and pathways affecting *in vitro* growth and fitness in minimal medium (MM) containing different single carbohydrate as the sole carbon source. About 2,000 Tn-mutants were screened for impaired growth in MM with one of the following carbon sources: glucose, fructose, mannose, mannitol, sucrose, glycerol, and glucose 6-phosphate (G6P). Impaired or abolished growth of *L. monocytogenes* was observed for twenty-one Tn-mutants with disruptions in genes encoding purine biosynthesis enzymes (*purL*, *purC*, *purA*, and *purM*), pyrimidine biosynthesis proteins (*pyrE* and *pyrC*), ATP synthase (*atpI* and *atpD2*), branched-chain fatty acids (BCFA) synthesis enzyme (*bkdA1*), a putative lipoprotein (LMOF2365_2387 described as *LP2387*), dUTPase family protein (*dUTPase*), and two hypothetical proteins. All Tn-mutants, except the *atpD2* mutant, grew as efficiently as wild-type strain in a nutrient rich media. The virulence of twenty-one Tn-mutants was assessed in mice at 72 h following intravenous (IV) infection. The most attenuated mutants had Tn insertions in *purA*, hypothetical protein (LMOf2365_0064 described as *HP64*), *bkdA1, dUTPase, LP2387*, and *atpD2*, confirming the important role of these genes in pathogenesis. Six Tn-mutants were then tested for ability to replicate intracellularly in murine macrophage J774.1 cells. Significant intracellular growth defects were observed in two Tn-mutants with insertions in *purA* and *HP64* genes, suggesting that an intact purine biosynthesis pathway is important for intracellular growth of *L. monocytogens.* These findings may not be fully generalized to all *of L. monocytogenes* strains due to their genetic diversity. In conclusion, Tn-mutagenesis identified that biosynthesis of purines, pyrimidines, ATP, and BCFA are important for *L. monocytogens* pathogenesis. Purine and pyrimidine auxotrophs play an important role in the pathogenicity in other bacterial pathogens, but our study also revealed new proteins essential for both growth in MM and *L. monocytogenes* strain F2365 virulence.

## Introduction

*Listeria monocytogenes* is a facultative intracellular pathogen that is responsible for listeriosis, a severe food-borne illness with a high mortality rate in immunocompromised people, children, older adults, and pregnant women (in whom it causes miscarriages, stillbirths or infection of newborns) (Vázquez-Boland et al., 2001; Lecuit, 2020). The route of infection in humans is usually through consumption of *L. monocytogenes*-contaminated food products, both processed and unprocessed, and the bacterium is frequently found and isolated from processed and ready-to-eat (RTE) food products (Radoshevich and Cossart, 2018). An increasing number of food product recalls have been issued recently due to adulteration with *L. monocytogenes* including ice cream, lettuce and/or packaged salads, fully cooked chicken, and deli meat (CDC, 2022). According to the Centers for Disease Control, about 1,600 people get sick from *L. monocytogenes* each year in the United States alone, with a mortality rate of 15–20%, making it one of the deadliest foodborne pathogens (CDC, 2011). For example, *L. monocytogenes* was responsible for the deaths of 10 people in Texas in 2010 due to consumption of chopped celery (Gaul et al., 2013) and 33 people in 28 different states in the US due to consumption of contaminated melons (CDC, 2016).

*L. monocytogenes* is regarded as a model species capable of growth in the environment, soil, food processing environments, and as a facultative intracellular pathogen of mammals (Anaya-Sanchez et al., 2021). A successful transition of *L. monocytogenes* from the extracellular to the intracellular environment requires sensing the surrounding environment and activating relevant metabolic pathways in a timely manner, especially with respect to carbon metabolism and utilization of various nutrients.

Upon encountering a host, *L. monocytogenes* expresses virulence factors responsible for host cell entry, escape from the entry vacuole, intracellular replication in the cytosol, and spread to systemic sites (Cahoon and Freitag, 2014). In the entry phagosomal vacuoles, intracellular *L. monocytogenes* have limited access to the host cells’ nutrients, while *L. monocytogenes* replicating in the cytosolic compartment have free access to the host cell nutrients (Chen and Pensinger, 2017). Thus, metabolic adaptations and utilization of varying externally available carbon sources and host-derived metabolites are essential for survival of *L. monocytogenes* and establishment of infection.

Much is known about virulence factors that are primarily regulated by transcriptional regulator PrfA (positive regulatory factor A) (Radoshevich and Cossart, 2018). However, little is known about *L. monocytogenes* metabolic adaptations and utilization of nutrients during listerial pathogenesis. The genome of *L. monocytogenes* reflects its ability to acquire and metabolize a range of different carbon sources, but previous isotopic labeling studies demonstrated that its intracellular metabolism relies mostly on glycerol and glucose 6-phosphate (G6P), which are generated in host cells (Eylert et al., 2008; Gillmaier et al., 2012). Glycerol is the primary carbon substrate for generation of energy and synthesis of some amino acids and fatty acids. G6P is an important carbon source for synthesis of sugar components essential for biosynthesis of the cell envelope and nucleotides (Grubmüller et al., 2014). A previous study showed that *L. monocytogenes* can utilize glucose-1-phosphate, G6P, fructose-6-phosphate, and mannose-6-phosphate as carbon sources and the uptake of these sugars is mediate by hexose phosphate transporter (Hpt), which is tightly controlled by PrfA (Ripio et al., 1997; Chico-Calero et al., 2002).

Carbon utilization and metabolism have been linked to pathogenicity in *L. monocytogenes* (Joseph et al., 2008; Muchaamba et al., 2019). Indeed, the availability of specific metabolites and sugars control the transcription, translation, and activation of PrfA and the expression of PrfA-dependent virulence genes. The objective of this study was to identify bacterial genes required for efficient growth and proliferation under limited nutrient conditions by screening for transposon (Tn) mutants that have a defect in growth in defined MM with different carbohydrates (glucose, fructose, mannose, mannitol, sucrose, and G6P, and glycerol, a non-carbohydrate carbon source. Tn-mutagenesis is a high-throughput technique used to identify determinants in a bacterial genome that are required for bacterial fitness (Fu et al., 2013). Using this method, we identified 21 Tn-mutants that have defective growth in MM with a sole carbon source. Further, some of these genes were evaluated for *L. monocytogenes* virulence and intracellular replication.

## Materials and methods

### Bacterial strains, media, and growth conditions

*Escherichia coli* (NF-E1130) harboring the pMC38 plasmid (Dr. Daniel Portnoy, UCLA) was propagated at 37°C in Luria-Bertani (LB) broth (Cao et al., 2007). *L. monocytogenes* wild-type F2365 strain (serotype 4b) was used as the parent strain for Tn mutagenesis (Nelson et al., 2004). The virulent F2365 strain was initially identified during a listeriosis outbreak in California in 1985, this outbreak was one of the deadliest foodborne outbreaks in the United States and serovar 4b represents the majority of the outbreak isolates (Melo et al., 2015). *L. monocytogenes* strains were grown at 37°C under aerobic conditions with shaking in brain heart infusion (BHI) broth or in chemically-defined minimal medium (MM) (Premaratne et al., 1991). For Tn-mutant screening, MM containing L-glutamine (4 mM) as nitrogen source and one of the following carbon sources was used: glucose (55.5 mM); fructose (25 mM); sucrose (125 mM); G6P (30 mM); mannose (20 mM); glycerol (41 mM); and maltose (30 mM). When appropriate, erythromycin (10 μg/ml), kanamycin (50 μg/ml), or chloramphenicol (30 μg/ml) was supplemented to the MM.

### Cell culture and media

Mouse fibroblast L cells (American Type Culture Collection, Manassas, Virginia) were propagated in ATCC-formulated Dulbecco’s Modified Eagle’s Medium (DMEM) supplemented with 10% fetal bovine serum (FBS). The murine macrophage cell line J774.1 was grown in DMEM supplemented with 10% FBS. Both the cell lines were maintained at 37°C and 5% CO_2_.

### Transposon mutagenesis

Transposon mutagenesis was performed with a mariner-based transposon pMC38 delivery vector following previously published methods (Cao et al., 2007; Azizoglu et al., 2014). Briefly, the electro-competent *L. monocytogenes* F2365 strain was transformed with pMC38 and selected on BHI agar supplemented with erythromycin and incubated at 30°C. Transformant positive colonies were grown overnight in BHI broth supplemented with kanamycin and erythromycin. Then the culture was diluted into fresh BHI medium supplemented with erythromycin and grown at 30°C for 1 hour. The temperature was increased to 42°C for ∼6 h until an OD_600_ of ∼0.5 was reached. These cultures were then diluted and spread on BHI agar with erythromycin and incubated at 42°C for transposon integration. Individual colonies from the Tn library were separately examined for kanamycin sensitivity to test for plasmid loss. Colonies resistant to only erythromycin were chosen for library construction and screening. For long-term storage before any phenotypic screening, approximately 2,000 Tn-insertion mutants were picked and inoculated in 96-well plates containing BHI broth (180 μl) and grown overnight at 37°C to an OD_600_ of approximately 1.0. Then glycerol (20 μl) was added to each well, and plates were stored at −80°C. *L. monocytogenes* wild-type was included in each plate as a positive control.

### Screening of transposon-mutants for growth-deficiency in defined minimal medium

For growth analysis in MM containing a single carbon source, 10 μl of Tn-insertion mutants (OD_600_ ≈1.0) from frozen libraries were transferred in duplicate into 96-well plates containing 90 μl of MM supplemented with one of the following carbon sources: glucose, mannose, fructose, sucrose, maltose, glycerol, or G6P. Following inoculation, 96-well plates were covered with Breathe-Easy film (Diversified Biotech) and incubated at 37°C in a Cytation 5 Cell Imaging Multi-Mode Reader (BioTek, Winooski, VT, United States). The OD_600_ for each Tn-mutant was recorded every hour for 48 h and compared to the OD_600_ of *L. monocytogenes* wild-type. A total of 21 Tn-mutants with reduced growth in 96-well plates containing MM were considered candidates and tested individually using culture tubes (four replicates) for further validation. Briefly, *L. monocytogenes* wild-type and Tn-mutants from overnight cultures (OD_600_ ≈1.0) were harvested by centrifugation and resuspended in PBS. After washing with PBS, bacterial suspension was used to inoculate 5 ml of MM with the respective carbon source at 1:100 dilution. The culture tubes were incubated at 37°C and the growth of each strain was determined by measuring OD_600_ after 48–72 h of incubation. Tn-mutants that grew on BHI but showed reduced or no growth in MM containing a single carbohydrate were selected for further analysis. For growth kinetics in BHI, overnight cultures of wild-type and Tn-mutants were normalized based on optical density at 600 nm (OD_600_) and used to inoculate fresh BHI broth at 1:100 dilution in a 24-well plate. Cytation 5 was used to monitor the growth by measuring OD_600_ every hour for 24-h. The growth assays were conducted in three independent experiments, and each experiment was run with four replicates.

### Determination of transposon-insertion sites

Transposon insertion sites of the mutants were determined as previously described (Cao et al., 2007; Azizoglu et al., 2014). Briefly, chromosomal DNA was isolated from the Tn-insertion mutants and used to amplify the fragments flanking the transposon through a nested arbitrary PCR reaction. The list of primers used in nested PCR are listed in Table 1. The 1*^st^* PCR amplification of the mutant genomic DNA was a touch-up PCR performed using two primer sets Marq207/Marq255 (PCR-1) and Marq207/Marq269 (PCR-2) using reaction conditions: (i) 95°C for 3 min; (ii) 15 cycles of 95°C for 30 s, 35°C for 30 s (increase 1°C every cycle), and 72°C for 3 min; and (iii) 15 cycles of 95°C for 30 s, 60°C for 30 s, and 72°C for 2 min. The products of the first reaction were used as template for a second PCR reaction with the following primer sets: Marq208/Marq256 for amplification of products from PCR-1 and Marq208/Marq270 for amplification of products from PCR-2. Standard reaction conditions were used for the second PCR: (i) 95°C for 3 min; (ii) 40°C cycles of 95°C for 30 s, 55°C for 30 s, and 72°C for 2 min; and (iii) 72°C for 5 min. To remove unincorporated nucleotides and primers prior to sequencing, PCR products were cleaned with ExoSAP-IT (Thermofisher) according to manufacturer instructions. The samples from PCR-1 were sequenced with Marq257, and PCR-2 were sequenced with Marq271. Sequence results were searched with BLAST analysis against the whole genome of *L. monocytogenes* strain F2365 (AE017262.2) from NBCI (threshold was ≥ 100% identity and *E*-value of 0).

**TABLE 1:** Bacterial strains and plasmids used for this study.

Bacterial strains, plasmid	Description	Source/ references
**Strains**		
*E. coli*		
S17-1	Competent cells	Cao et al., 2007
*L. monocytogenes*		
F2365	Wild-type serotype 4b strain	Nelson et al., 2004
F2365-pMC38	F2365 harboring plasmid pMC38; Em^R^, Kan^R^	This study
**Plasmids**		
pMC38	Mariner-based transposon delivery plasmid	Cao et al., 2007
**Primers for deletion strain and complementation (5′–3′)**		
Marq207	GGCCACGCGTCGACTAGTAC NNNNNNNNNNGTAAT	1st PCR arbitrary
Marq208	GGCCACGCGTCGACTAGTAC	2nd PCR
Marq255	CAGTACAATCTGCTCTGATG CCGCATAGTT	1st PCR Tn
Marq256	TAGTTAAGCCAGCCCCGACA CCCGCCAACA	2nd PCR Tn nested
Marq257	CTTACAGACAAGCTGTGACC GTCT	Sequencing
Marq269	GCTCTGATAAATATGAACATG ATGAGTGAT	1st PCR Tn
Marq270	TGTGAAATACCGCACAGA TGCGAAGGGCGA	2nd PCR Tn nested
Marq271	GGGAATCATTTGAAGGTT GGTACT	Sequencing
Marq206	TGTCAGACATATGGGCACAC GAAAAACAAGT	Southern blot probe
Marq254	CGTGGAATACGGGTTTGCT AAAAG	Southern blot probe

### Southern blot hybridization

Southern blot hybridization for six Tn-mutants was performed by Cel*plor*, a molecular biology CRO company^1^ to confirm the accuracy of the mapping methodology. Genomic DNA of mutants was digested with *EcoR*I (New England Biolabs, Beverly, MA, USA) overnight, resolved on agarose gels, and transferred to a nylon membrane. A 400 bp PCR product generated using primers Marq206/254 and pMC38 was used as a probe for the hybridization to locate the inserted DNA in targeted disrupted mutants.

### Mutant virulence

All animal experiments were approved by the Institutional Animal Care and Use Committee of Mississippi State University (protocol # IACUC-21-461). Eight-week-old female Swiss Webster mice were obtained from Charles River laboratories (Wilmington, MA, USA) and housed at a rate of 5 mice per cage. Twenty-one Tn-mutants were tested for virulence. Mice were placed in quarantine for 3 days before they were released for the study. Overnight cultures of wild-type and Tn-mutants grown in BHI broth were standardized based on OD_600_, washed, resuspended in sterile saline. Serial dilutions were spread on agar plates for estimation of colony forming units (CFU). Mice (5 mice per group) were injected intravenously (IV) into a tail vein with 200 μL of the diluted bacterial suspension, achieving infectious dose of 2 x10^4^ CFU/mL. The negative control group received only saline, and the positive control group received *L. monocytogenes* wild-type. Mice were frequently monitored for signs of clinical deterioration. Mice were euthanized 72-h post infection, and their livers and spleens were harvested, followed by homogenization in sterile PBS. Dilutions of the homogenized suspension were spread on BHI agar and incubated at 37°C. Colonies were counted after 48 h, and CFU/g of tissue was calculated to analyze the virulence of each strain.

### Intracellular replication in murine macrophages

The ability of *L. monocytogenes* mutants to invade and replicate within host cell cytosol was compared to the wild-type strain in the presence and absence of gentamicin as previously described (Alonzo and Freitag, 2010). Confluent 12-well plates containing J774.1 murine macrophage cells were infected with the wild-type and Tn-mutants at a multiplicity of infection (MOI) of 1 bacterium to 1 macrophage. The infected plates were centrifuged and incubated for 1 h at 37°C and 5% CO_2_. Then the media was removed, and the cells were washed three times with sterile PBS. To measure total counts of bacteria associated with macrophages without gentamicin treatment (zero time points), monolayer cells were lysed with 0.5% Triton X-100 for 10 min followed by spreading on BHI agar to calculate CFU (Abdelhamed et al., 2015). To monitor intracellular survival and replication inside the macrophages, DMEM containing gentamicin (10 μg/ml) was added to each well and incubated at 37°C and 5% CO_2_ for 2, 4, 6, 24–h. At the end of each time point, media was removed, and the monolayer cells were washed. Cells were dislodged by adding trypsin and scraping. Serial dilutions of the lysed cell suspensions were spread on BHI agar and incubated at 37°C. Bacterial colonies were counted after 48 h, and CFU/ml was calculated for the wild-type and Tn-mutants for each time point. Bacterial intracellular growth and replication were performed in two independent experiments with three replicates.

### Cell-to-cell spread

The ability of Tn*-*mutants to replicate in the host cytosol and spread from cell to cell was evaluated in plaque assays as described (Abdelhamed et al., 2020). Briefly, L2 fibroblast cells were grown in 6-well plates. Overnight cultures of wild-type and Tn-mutants were used to infect the L2 fibroblast cells at an MOI of 1–1. Plates were incubated for 1 h at 37°C and 5% CO_2_. After 1 h, the infected cells were washed and overlaid with a mixture containing DMEM and 1% Sea Plaque agarose supplemented with gentamicin (10 μg/ml). After solidification of the agarose, the plates were incubated at 37°C and 5% CO_2_ for 4 days. Staining of the plaques was performed by adding a second overlay of the DMEM and 1% Sea Plaque agarose mixture containing 0.01% Neutral red (Sigma Aldrich, St. Louis, MO). The plates were stained overnight at 37°C. Plaques sizes were measured using Olympus cellSens software (Olympus Corp. Shinjuku City, Tokyo, Japan). Experiments were repeated twice, and a minimum of 10 plaques were measured to determine plaque size.

### Statistical analysis

Statistical significance was assessed using the Kruskal-Wallis test with GraphPad Prism 5 software (GraphPad Software San Diego, CA) to compare bacterial concentrations in mice tissues infected with wild-type and Tn-mutants. Statistical significance in intracellular replication was analyzed by two-way ANOVA (multiple groups and different time points) and Dunnett’s multiple comparison test. Statistical significance of mutants’ adhesion/replication to macrophages at zero time point was analyzed by one-way ANOVA and Dunnett’s multiple comparison test. All other data were analyzed with Student’s *t*-test. Data are presented as the mean ± SEM. A *P* value of less than 0.05 was considered significant.

## Results

### Identification of transposon-mutants deficient in growth in minimal medium

A library consisting of ∼2,000 Tn-mutants was screened to identify mutants with impaired growth in MM in the presence of one of the following carbon sources: glucose, mannose, fructose, sucrose, maltose, glycerol, or G6P. The screening identified 21 Tn-mutants that exhibited a significant reduction in growth rate compared to the wild-type in at least one of the media tested. The percentage growth of the identified candidates compared to wild-type in the presence of different carbon sources is presented in Table 2. The average OD_600_ values of 21 Tn-mutants are listed in Supplementary Table 1. Southern blotting confirmed the presence of a single Tn-insertion in the genome of the selected mutants.

**TABLE 2:** Tn-insertion mutants with defective growth in MM containing glucose (Glu), fructose (Fru), mannose (Man), sucrose (Suc), maltose (Malt), glycerol (Gly), or glucose 6-phosphate (G6P) as the sole carbon source.

				Percent growth in MM supplemented with respective carbon source compared to the wild-type						
				
	Mutant name	Locus tag*	Annotation	Glu	Fru	Man	Suc	Malt	Gly	G6P
Purine metabolism	*purL*::Tn	LMOf2365_1794	Phosphoribosylformylglycinamidine synthase I I	36.7	37.5	23.5	11.7	24.2	15.9	39.1
	*purM*::Tn	LMOf2365_1792	Phosphoribosylformylglycinamidine cyclo-ligase	28.5	40.95	30.4	11.2	23.9	16.57	29.03
	*purA*::Tn	LMOf2365_0065	Adenylosuccinate synthetase	27.2	34.16	39.6	7.67	17.8	15.98	31.65
	*purC*::Tn	LMOf2365_1797	Phosphoribosylaminoimidazole-succinocarboxamide	31.7	35.75	31.1	15.8	29.09	18.52	31.85
Pyrimidine metabolism	*pyrE*::Tn	LMOf2365_1859	Orotate phosphoribosyltransferase	14.2	11.76	13.5	21.5	10.6	9.55	12.50
	*pyrC*::Tn	LMOf2365_1865	Dihydroorotase, multifunctional complex type	6.00	15.61	19.9	4.08	10.8	15.59	10.69
ATP synthase protein	*atpI*::Tn	LMOf2365_2509	ATP synthase protein I	68.7	67.42	88.8	52.52	13.59	49.51	60.89
	*atpD2*::Tn	LMOf2365_2502	ATP synthase F1, beta subunit	77.0	88.69	80.5	78.9	12.7	53.41	78.43
	*2814*::Tn	LMOf2365_2814	Hypothetical protein	110	105.	114	57.79	42.04	51.07	78.43
	*HP64*::Tn	LMOf2365_0064	Hypothetical protein	70.2	54.75	71.7	16.0	40.3	25.34	48.59
	*manA*::Tn	LMOf2365_2143	Mannose-6-phosphate isomerase, class I	109	101	8.08	88	51.3	50.68	73.39
	*proA*::Tn	LMOf2365_1276	Glutamate-5-semialdehyde dehydrogenase	67.2	72.85	100	53.2	74.7	49.71	94.56
	*2641*::Tn	LMOf2365_2641	Ribulose-phosphate 3-epimerase family protein	82.2	74.43	104	90.89	56.90	53.61	74.40
	*gcvT*::Tn	LMOf2365_1365	Glycine cleavage system T protein	117	102	121	115.5	20.81	51.07	84.68
	*LP2387*::Tn	LMOf2365_2387	Putative lipoprotein	121.75	102	0.48	80.34	63.69	58.48	106.65
	*dUTPase*::Tn	LMOf2365_1715	dUTPase family protein	72.5	57.69	61.2	23.74	36.94	31.38	33.06
	*bkdA1*::Tn	LMOf2365_1389	2-oxoisovalerate dehydrogenase E1 component	61.75	71.49	59.8	30.46	41.61	14.23	5.04
	*pdxS*::Tn	LMOf2365_2133	Pyridoxine biosynthesis protein	42.25	37.56	34.6	12.47	21.87	18.71	28.63
	*hom*::Tn	LMOf2365_2520	Homoserine dehydrogenase	58.50	59.95	62.23	15.35	32.91	30.21	44.96
	*pheA*::Tn	LMOf2365_1555	Prephenate dehydratase	105	134.1	116	81.2	64.3	50.10	91.13
	*0800*::Tn	LMOf2365_0800	Pseudogene	59.25	40.05	43.9	14.3	0.42	25.7	41.3

*Insertion sites.

Among the list of identified Tn-mutants are four mutants with insertion sites located in genes encoding purine biosynthesis enzymes, including *purL*::Tn, *purC*::Tn, *purA*::Tn, and *purM*::Tn. In addition, two mutants have Tn-insertion in genes encoding pyrimidine biosynthesis enzymes, including *pyrE*::Tn and *pyrC*::Tn. Two of the Tn-mutants have insertions in genes encoding ATP synthase, *atpI*::Tn and *atpD2*::Tn. One mutant had a Tn-insertion in a gene encoding 2-oxoisovalerate dehydrogenase E1 component, α subunit of the branched-chain alpha-keto acid dehydrogenase (BKD) complex. Two mutants had Tn-insertions in genes annotated as hypothetical proteins (LMOf2365_2814 and LMOf2365_0064), and one mutant had a Tn-insertion in a gene annotated as a pseudogene (LMOf2365_0800). One transposon mutation mapped to LMOF2365_2387, a putative lipoprotein.

### Growth of *atpD2*::Tn mutant is affected in a nutrient rich media

**FIGURE 1:**
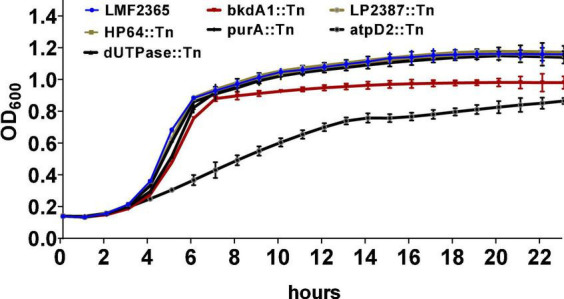
Growth of the Tn-mutants and wild-type *L. monocytogenes* in BHI medium at 37°C for 24 h. The growth kinetic was monitored using optical density measurements at 600 nm. Data represent he means ± standard error of three independent experiments with four replicates.

To understand the growth kinetics of the Tn-mutants, their growth rate in BHI was determined over a 24-h period at 37°C. The *atpD2*::Tn mutant had marked reduction in the growth with a doubling time of 190.9 min compared to the wild-type that doubled in 84.2 min (Figure 1). The doubling time of *dUTPase*::Tn and *bkdA1*::Tn mutants slightly increased by 9.9 and 2.9 min, respectively, compared to wild-type but this difference was not significant. In stationary phase, *bkdA1*::Tn did not achieve the same peak density as the parental strain. All other Tn-mutants grew as well as wild-type strain in BHI rich media. The growth defect in *HP64*::Tn and *purA*::Tn mutants could be restored to levels similar to that obtained for the parent strain by addition of adenine to MM (data not shown).

### Mutants with impaired growth in minimal medium display varied attenuation in mice

Because pathogenesis of *L. monocytogenes* requires metabolic adaptation and nutrient uptake, we determined whether the MM growth defective Tn-mutants are attenuated *in vivo* using murine model.

**FIGURE 2:**
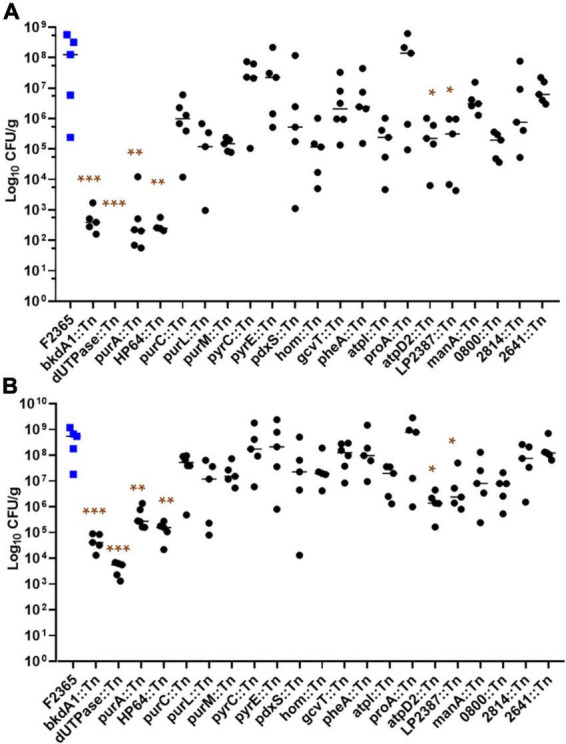
Virulence phenotypes of *L. monocytogenes* Tn-insertion mutants. Each animal group (5 mice per cage) was intravenously infected with 2 × 10^4^ colony forming units (CFUs) with the indicated strains. At 72 h post-infection, bacterial concentrations were determined for livers **(A)** and spleens **(B)**. Each point represents one mouse. Statistical analysis was performed with Kruskal-Wallis test in GraphPad Prism. *Indicates a significant difference at *P* < 0.05, **indicates a significant difference at *P* < 0.001, and ***indicates a significant difference at *P* < 0.0001.

Bacterial concentrations in liver and spleen tissues after 72 h of infection are presented in Figure 2. We detected a reduction in bacteria concentration in several Tn-mutants, but the reduction was only statistically significant in *purA*::Tn, *HP64*::Tn, *bkdA1*::Tn, *dUTPase*::Tn, *LP2387*::Tn, and *atpD2*::Tn. Log_10_ CFU/g changes in bacterial burden of the tested Tn-mutants are summarized in Table 3.

**TABLE 3:** Log_10_ CFU/g changes in concentration of Tn-mutants compared to wild-type in the liver and spleen of infected mice.

Tn-mutants	Liver	Spleen
*purA*::Tn	4.88**	2.95**
*purC*::Tn	1.98	0.9
*purL*::Tn	2.87	1.29
*purM*::Tn	3.05	1.22
*pyrC*::Tn	0.67	0.05
*pyrE*::Tn	0.49	0.19
*HP64*::Tn	5.82**	3.47**
*bkdA1*::Tn	5.1***	3.8***
*dUTPase:*:Tn	8.3***	5***
*LP2387*::Tn	2.66*	1.63*
*pdxS::*Tn	0.84	0.57
*hom*::Tn	2.81	0.94
*gcvT*::Tn	1.34	0.48
*pheA*::Tn	1.17	0.08
*atpD2*::Tn	2.7*	2.4*
*atpDI*::Tn	2.69	1.37
*proA*::Tn	0.37	0.13
*2814*::Tn	1.064	0.65
*2641*::Tn	1.29	0.36
*0800*::Tn	2.66	1.63
*manA*::Tn	3.03	1.81

The values were calculated by subtracting mean log_10_ CFU/g of wild-type infected animals from mean of animals infected with Tn-mutants. *Indicates a significant difference at *P* < 0.05, **indicates a significant difference at *P* < 0.001, and ***indicates a significant difference at *P* < 0.0001.

**FIGURE 3:**
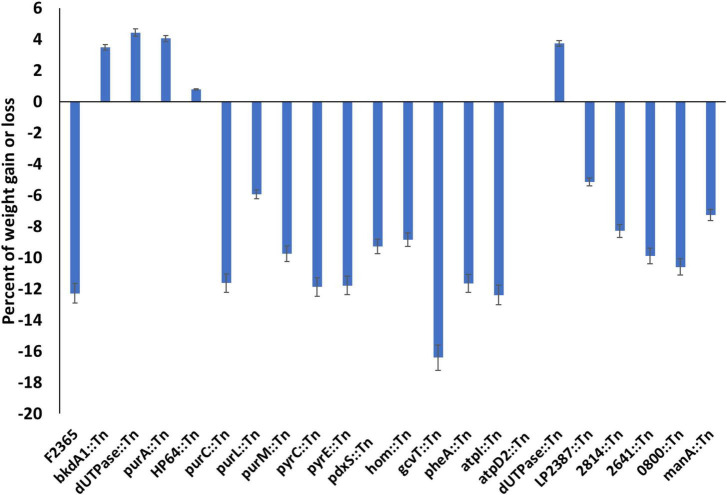
Average changes in body weight in mice infected with Tn-mutants compared to mice infected with wild-type *L. monocytogenes*. Variation in body weight (gain or loss) was calculated by subtracting body weight of each mouse at end of experiment from the baseline body weight at the time of the injection. Values represent the mean of 5 mice.

The change in the body weight of the mice from the time prior to challenge and at the time of euthanasia reflected the severity of infection caused by each Tn-mutant (Figure 3). The mice infected with *purA*::Tn, *HP64*::Tn, *bkdA1*::Tn, and *dUTPase*::Tn mutants showed significant weight gain compared to mice infected with wild-type. The mice group challenged with *atpD2*::Tn neither gained any significant weight nor lost any during their 8 days of incubation with bacteria. However, the mice infected with *purC*::Tn, *purL*::Tn, *purM*::Tn, *pyrC*::Tn, *pyrE*::Tn, *pdxS*::Tn, *hom*::Tn, *gcvT*::Tn, *pheA*::Tn, and *atpI*::Tn showed reduction in body weight similar to mice infected with wild-type.

### Attenuated transposon-mutants showed a defect in cellular invasion, intracellular replication and/or cell-to-cell spread

**FIGURE 4:**
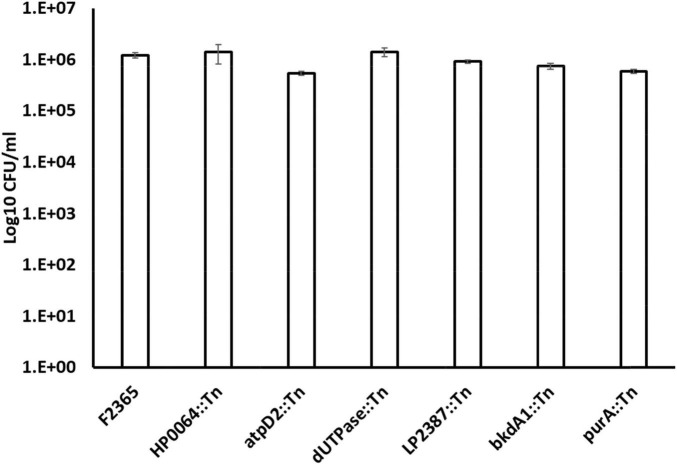
The total number of CFU/ml of wild-type *L*. *monocytogenes* strain F2365 and Tn-mutant strains obtained without gentamicin treatment to J774.1 murine macrophage cells at 1 h post-infection. Numbers on the Y axis indicate bacterial quantities (CFU/ml). Statistical analysis did not show any significant differences (*P* > 0.05). The experiment was repeated twice with three replicates each.

The first steps of *L. monocytogenes* pathogenesis rely on the ability of the bacteria to invade the host cells, escape the vacuole, replicate within the nutrient-rich host cell cytosol, and move from one cell to another without exposing itself to the extracellular environment. We determined the ability of six Tn-mutants (*purA*::Tn, *HP64*::Tn, *dUTPase*::Tn, *bkdA1*::Tn, *LP2387*::Tn, and *atpD2*::Tn) for cellular invasion, intracellular replication, and cell-cell spread in comparison to the wild-type. These six mutants were selected because they showed significant attenuation in mice. By 1-h post-infection and without gentamicin treatment, there was no significant difference (*P* > 0.05) in the total number of CFU between the six mutants compared to the wild-type (Figure 4).

**FIGURE 5:**
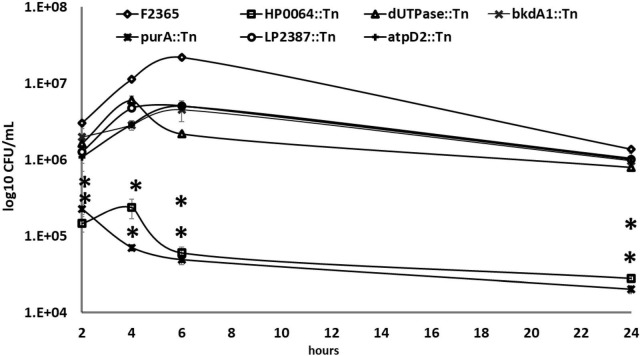
Intracellular growth of Tn-mutants compared to the wild-type *L. monocytogenes* strain in J774 macrophage-cells using an MOI of 10:1. At 2, 4, 6, and 24 h pot-infection, cells were lysed, and CFUs were enumerated. The experiment was repeated twice with three replicates each. Error bars are the standard error of mean. Two-way ANOVA and Dunnett’s multiple comparison test were used to measure the statistical significance between Tn-mutants and wild-type at different time points. Asterisks (*) indicate statistically significant differences (*P* < 0.05).

We next determined intracellular replication of the Tn-mutants by gentamicin protection assays in J774.1 murine macrophages at 2-, 4-, 6-, and 24-h post-infection (HPI). The *purA*::Tn and *HP64*::Tn mutants exhibited significant deficiency to replicate within J774.1 macrophages compared to the wild-type strain (Figure 5). The reduction in the intracellular replication of *purA*::Tn and *HP64*::Tn strains was 1.13 and 1.32-log_10_ at 2 h, 2.21 and 1.68-log_10_ at 4 h, 2.65 and 2.56-log_10_ at 6 h, and 1,83 and 1.68-log_10_ at 24 h compared to the wild-type strain, respectively. At 2-, 4-, 6-, and 24-h after infection, the intracellular bacterial numbers of the *atpD2*::Tn mutant were lower than those of the wild-type strain with log_10_ reduction of 0.45, 0.59, 0.64, and 0.12.

The *dUTPase*::Tn mutant showed a substantial reduction of 0.27-log_10_, 027-log_10_, 1-log_10_, 0.23-log_10_ in the CFU recovered from infected macrophages compared to those infected with the wild-type after 2, 4, 6, and 24 h of infection. The intracellular replications of *bkdA1*::Tn and *LP2387*::Tn were almost indistinguishable from those of the wild-type strain with a log_10_ reduction of 0.18 and 0.38 at 2 HPI, 0.60 and 0.38 at 4 HPI, and 0.68 and 0.63 at 6 HPI, and 0.15, 0.13, and 0.12 at 24 HPI compared to the wild-type, respectively.

**FIGURE 6:**
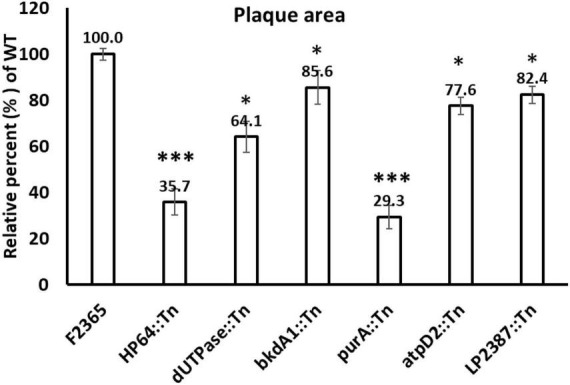
Plaque formation of wild-type *L. monocytogenes* and Tn-mutants in monolayers of murine L2 fibroblast cells. The mean plaque size of each strain is shown as a percentage relative to the wild-type plaque size. Error bars represent standard deviations of the mean plaque size from two independent experiments. Statistical analysis was performed using Student’s *t*-test. Asterisks indicate statistically significant differences (*P < 0.05, ***P < 0.001).

To assess the ability of the Tn-mutants to spread from cell to cell, a plaque assay in L2 fibroblast cells was conducted for six Tn-mutants, and plaque diameters were compared to wild-type. The plaque area formed by *HP64*::Tn, *purA*::Tn, *dUTPase*::Tn, *bkdA1*::Tn, *atpD2*::Tn, and *LP2387*::Tn mutants was significantly smaller compared to the wild-type, measuring 35.73, 29.3, 64.1, 85.58, 77.55, and 82.64% of wild-type plaque diameter, respectively (Figure 6). The small plaque-phenotype was the most prominent in the *purA*::Tn and *HP64*::Tn mutants where the plaque sizes were approximately 30% of the wild-type F2365, reflecting that *purA* and *HP64* are required not only for cell-to-cell spread, but also for intracellular survival and growth.

## Discussion

*Listeria monocytogenes* fitness during pathogenesis requires efficient utilization of available nutrients. Furthermore, the function of PrfA, the master regulator of virulence genes in *L. monocytogenes*, is dependent on the metabolic state of the bacterium. Carbon metabolism is among several metabolic pathways that has been associated with functional state of PrfA; glucose and other sugars are known to inhibit PrfA activity, but metabolism of non-carbohydrate sources such glycerol activates PrfA (Mertins et al., 2007; Chen et al., 2017). However, our understanding of nutritional signaling, metabolism, and virulence in *L. monocytogenes* is still incomplete.

To further bridge this knowledge gap, we conducted a genetic screening and isolated insertional mutants that are deficient in growth in MM supplemented with different carbohydrate substrates but can grow in BHI (nutrient rich media). Growth in MM requires the biosynthesis of several metabolites that can be scavenged from a complex growth media. We identified 21 Tn-mutants that show growth deficiency when grown in MM supplemented with various carbon sources. We have characterized the mutants and their role in establishing infection.

By Tn-mutagenesis, we defined four genes encoding purine biosynthesis enzymes which are essential for *L. monocytogenes* in growth in MM. The products of these genes are phosphoribosylformyl glycinamidine synthase subunit (*purL*), phosphoribosylformylglycinamidine cyclo-ligase (*purM*), phosphoribosylaminoimidazole-succinocarboxamide synthase (*purC*), and adenylosuccinate synthetase (*purA*). In addition, *HP64*::Tn has Tn-insertion in a hypothetical protein (LMOf2365_0064) that is 68 bp upstream of *purA* (LMOf2365_0065). The growth defect in *HP64*::Tn and *purA*::Tn mutants could be restored by addition of adenine to MM. Thus, the phenotypic effect shown by *HP64*::Tn is apparently due to a defect in the downstream *purA* gene or may be *HP64* has its own function in purine biosynthesis.

Interestingly, Tn-mutants for purine and pyrimidine biosynthesis showed severely impaired growth in MM; however, the mutants grew as well as wild-type bacteria in BHI broth. These findings are typical for purine and pyrimidine auxotrophs. Because they are unable to synthesize purines or pyrimidines, the respective auxotrophic strains must rely on salvage of nutrients, which are not readily available in MM, but they are available in nutrient-rich BHI medium.

PurA catalyzes the conversion of inosine mono phosphate to adenylosuccinate in the purine metabolic pathway for the synthesis of adenine. Purine auxotrophs of several bacterial pathogens are attenuated. For example, the following mutants are all attenuated: *purA* and *purB* mutants of *L. monocytogens* (Faith et al., 2012); *purF, purG, purC*, and *purA* mutants in *Salmonella* (McFarland and Stocker, 1987), *purL* mutant in *Brucella abortus* (Alcantara et al., 2004), and *purE* mutant in *B. melitensis* (Crawford et al., 1996). Purine biosynthesis is indispensable for *Staphylococcus aureus* growth in human blood and *in vivo* pathogenicity (Connolly et al., 2017).

In the present study, we observed a critical role of *purA* in intracellular multiplication and cell to cell spread of *L. monocytogenes* within infected host cells. The reduced plaque sizes and intracellular replication in the *purA*::Tn mutant was partially restored when the media was supplemented with adenine (data not shown). This finding suggests that adenine is not available for listerial salvage in the intracellular environment. Several research groups have reported a reduced ability of a *purA* Tn-mutant to multiply intracellularly and within intestinal epithelial cells (Sauer et al., 2011; Faith et al., 2012).

Our screening of Tn-mutants identified the gene coding for 2-oxoisovalerate dehydrogenase E1 component, α subunit of the branched-chain alpha-keto acid dehydrogenase (BKD) complex, which is a necessary enzyme for branched-chain fatty acids (BCFA) synthesis (Giotis et al., 2007; Sun and O’Riordan, 2010; Sun et al., 2012). BCFA represent more than 90% of the membrane composition in *L. monocytogenes*, and modulating BCFA membrane composition is a critical mechanism for regulating membrane fluidity to allow adaptation to low temperatures (Annous et al., 1997; Zhu et al., 2005; Giotis et al., 2007). The importance of BKD enzymes in bacterial virulence has been well characterized in several pathogens (Annous et al., 1997; Zhu et al., 2005; Giotis et al., 2007). In *L. monocytogenes*, Tn-mutants in BKD encoding genes *ipd* and *bkdB* (*cld-1* and *cld-2* mutants) exhibited defects in BCFA composition, cold sensitivity, and a significantly less-fluid membrane than the parent strain (Jones et al., 2002). In addition, *L. monocytogenes cld-1* and *cld-2* mutants exhibit lower tolerance to acidic and alkaline pH than parent strain (Giotis et al., 2007).

In our study, we demonstrated that inactivation of *bkdA1* resulted in complete attenuation of virulence in mice. The mice infected with *bkdA1*::Tn exhibited no signs of morbidity and had low concentrations of bacteria in spleen and liver. This phenotypic effect may be due to a reduction in anteiso-BCFAs, which provide fluidity to the listerial membrane. We showed that a *bkdA1*::Tn mutant was defective for growth in MM supplemented with glucose, sucrose, maltose, glycerol, or G6P. In BHI broth, the *bkdA1*::Tn mutant showed no significant difference in doubling time compared to the wild-type at 37°C. Our findings indicate that MM does not support the scavenging of an exogenous nutrient necessary for growth of a BCFA auxotrophic strain.

Deoxyuridine triphosphatase (dUTPase) hydrolyzes dUTP to dUMP (deoxyuridine monophosphate) and pyrophosphate (Bessman et al., 1958). It is an important enzyme that protects DNA against uracil incorporation (Dubois et al., 2011). Through catalytic conversion of dUTP to dUMP, dUTPase also provides precursors for synthesis of thymidine nucleotides essential in DNA replication. It also maintains a low cellular concentration of dUTP, thus preventing its incorporation into DNA (Vértessy and Tóth, 2009). In *E. coli* and yeast, mutation of dUTPase is lethal (el-Hajj et al., 1988; Gadsden et al., 1993). In the current study, a *L. monocytogenes dUTPase*::Tn mutant displayed a 30–40% reduction in growth compared to the wild-type in MM with glucose, fructose, and mannose. Its growth was more affected in MM with sucrose, maltose, glycerol, and glucose 6-phosphate (65–80% reduction compared to the wild-type). When J774.1 macrophage cells were infected with the *dUTPase*::Tn mutant, the number of bacteria reached a peak at 4 h followed by a decline in intracellular replication. These results were substantiated by plaque assay that indicated a 60% reduction in plaque number and size compared to wild-type, indicating a defect in intracellular replication and/or cell-to-cell spread. Mice infected with *dUTPase*::Tn cleared the bacteria completely from liver after 72 h of infection with a 5-log reduction in viable bacterial cells observed in spleen. Further investigation will focus on the mechanisms that allow the *dUTPase*::Tn mutant to grow in nutrient rich media but prevent their proliferation in mice with an intact immune system.

Adenosine triphosphate synthase beta 2 subunit (*atpD2*) is a part of the plasma membrane ATP synthase complex catalytic core (F_1_), which catalyzes the phosphorylation of ADP using inorganic phosphate to form ATP at the expense of a proton gradient across the membrane (Revington et al., 2002). *L. monocytogenes* also generates ATP through substrate level phosphorylation that occurs during the glycolytic pathway (Sauer et al., 2019). In the present study, inactivation of *atpD2* gene significantly reduced the doubling time of *L. monocytogenes* F2365 strain in nutrient rich BHI media, indicating the requirement of *atpD2* gene for efficient replication of *L. monocytogens*. This is likely due to an impairment in ATP synthesis and resulting insufficient ATP generation for aerobic growth (Müller-Herbst et al., 2014). In our study, the *atpD2*::Tn mutant was grown under aerobic conditions, and its anaerobic growth dynamics remains to be determined. The *atpD2*::Tn mutant was also defective for growth in mice liver and spleen, suggesting that generation of ATP via substrate level phosphorylation alone is not sufficient for *L. monocytogenes* replication in the host.

The *LP2387*::Tn mutant has a mutation mapped to LMOF2365_2387, which encodes a putative lipoprotein. Lipoproteins are a major category of bacterial surface proteins that have diverse functions and often have important effects on pathogen/host interactions (García-del Portillo and Cossart, 2007; Machata et al., 2008). Up to 68 putative lipoproteins have been predicted in the genome of *L. monocytogenes* include ABC transporter lipoproteins for acquisition of micronutrients (Baumgärtner et al., 2007). In the present study, the *LP2387*::Tn mutant showed a decreased capacity to colonize in mice and displayed growth defect in MM. Mutation of individual lipoproteins affects *Streptococcus pneumoniae* sensitivity to environmental stress, adhesion to host tissues, and interactions with host phagocytes (Tseng et al., 2002). Deletion of the *S. pneumoniae lgt* results in reduced growth in physiological media with glucose, sucrose, raffinose, or maltotriose as the only carbon source (Chimalapati et al., 2012). Our result suggested that *LP2387* may be part of ABC transporter lipoproteins that play a role in uptake of micronutrients for the growth and survival of *L. monocytogenes* F2365 strain in the host.

In summary, we report the construction and screening of a Tn-mutant library in *L. monocytogenes* to identify genes essential for growth under limited nutrient conditions. These include genes necessary for purine biosynthesis, pyrimidine biosynthesis, and BCFA metabolism, and ATP synthase protein. Purine biosynthesis and BCFA synthesis mutants were also defective for *L. monocytogens* virulence. This work indicates that *L. monocytogenes* metabolism affects its ability to cause disease and replicate in the host, and it provides evidence for the pathogen’s need to salvage specific nutrients during infection. Finally, our findings may not be fully generalized to all *L. monocytogenes* strains due to genetic and phenotypic diversity between strains. Further work will focus on the construction of clean deletion mutants and complementation strains corresponding to 21 genes identified in the present study and testing their ability to grow in MM and virulence in a mouse model of *L. monocytogenes* infection. Although intravenous infection is commonly used as a murine model to characterize the pathogenicity of *L. monocytogenes* strains it completely bypasses the gastrointestinal phase of the infection and bloodstream invasion. While sharing similarities, mouse and human macrophage cells exhibit numerous differences in microbial responses (Murray and Wynn, 2011). These differences must be considered when extrapolating mouse results to humans. Thus, evaluating the survival and replication of the Tn-mutants in human macrophage are warranted.

## Data availability statement

The original contributions presented in this study are included in the article/Supplementary material, further inquiries can be directed to the corresponding author.

## Ethics statement

All animal experiments were done in accordance with a protocol (18-508) approved by the Institutional Animal Care and Use Committee at Mississippi State University.

## Author contributions

LN: library construction, data analyses, and writing first draft. OO: mutants screening and mice experiment. NA: cell cultures. RR: mice experiment. ML: experimental design, review, and edit. HA: experimental design, library construction, data analysis, review, and edit. All authors wrote and approved the manuscript.

## Footnotes

1
www.celplor.com
