# Supplementary material for: Identification of genetic elements required for Listeria monocytogenes growth under limited nutrient conditions and virulence by a screening of transposon insertion library
Source: Front Microbiol. 2022 Oct 13;13:1007657. doi: 10.3389/fmicb.2022.1007657 (PMC9608667; doi:10.3389/fmicb.2022.1007657)
Supplement: Supplementary file 1 [file Table_1.docx]

**Supplementary table 1.** Optical density (OD_600_) of Tn-mutants and wild-type in MM containing glucose, fructose, mannose, sucrose, maltose, glycerol, or G6P as the sole carbon source after 48-72 hours of growth in culture tubes. Data are averages ± SEM of two experiments with four replicates.

| **Name** | **Glucose** | **Fructose** | **Mannose** | **Sucrose** | **Maltose** | **Glycerol** | **G6P** |
| --- | --- | --- | --- | --- | --- | --- | --- |
| **F2365** | 0.4±0.04 | 0.442±0.05 | 0.421±0.04 | 0.417±0.03 | 0.471±0.04 | 0.513±0.05 | 0.496±0.04 |
| ***purL*::Tn** | 0.147±0.04 | 0.166±0.04 | 0.099±0.04 | 0.049±0.02 | 0.114±0.03 | 0.082±0.03 | 0.194±0.04 |
| ***purM*::Tn** | 0.114±0.04 | 0.181±0.04 | 0.128±0.04 | 0.047±0.04 | 0.113±0.04 | 0.085±0.04 | 0.144±0.04 |
| ***purA*::Tn** | 0.109±0.04 | 0.151±0.05 | 0.167±0.03 | 0.032±0.04 | 0.084±0.04 | 0.082±0.04 | 0.157±0.04 |
| ***purC*::Tn** | 0.127±0.04 | 0.158±0.04 | 0.131±0.05 | 0.066±0.04 | 0.137±0.04 | 0.095±0.04 | 0.158±0.04 |
| ***pyrE*::Tn** | 0.057±0.04 | 0.052±0.04 | 0.057±0.02 | 0.09±0.04 | 0.05±0.04 | 0.049±0.04 | 0.062±0.04 |
| ***pyrC*::Tn** | 0.024±0.04 | 0.069±0.04 | 0.084±0.01 | 0.017±0.04 | 0.051±0.04 | 0.08±0.04 | 0.053±0.04 |
| ***atpI*::Tn** | 0.275±0.04 | 0.298±0.04 | 0.374±0.04 | 0.219±0.04 | 0.064±0.04 | 0.254±0.04 | 0.302±0.04 |
| ***atpD2*::Tn** | 0.308±0.04 | 0.392±0.04 | 0.339±0.04 | 0.329±0.04 | 0.06±0.04 | 0.274±0.04 | 0.389±0.04 |
| ***2814*::Tn** | 0.443±0.04 | 0.467±0.04 | 0.482±0.07 | 0.241±0.04 | 0.198±0.04 | 0.262±0.04 | 0.389±0.04 |
| ***HP64*::Tn** | 0.281±0.05 | 0.242±0.04 | 0.302±0.08 | 0.067±0.04 | 0.19±0.04 | 0.13±0.04 | 0.241±0.04 |
| ***manA*::Tn** | 0.437±0.09 | 0.447±0.07 | 0.034±0.02 | 0.37±0.04 | 0.242±0.04 | 0.26±0.03 | 0.364±0.03 |
| ***proA*::Tn** | 0.269±0.04 | 0.322±0.06 | 0.422±0.05 | 0.222±0.04 | 0.352±0.03 | 0.255±0.04 | 0.469±0.08 |
| ***2641*::Tn** | 0.329±0.06 | 0.329±0.07 | 0.442±0.04 | 0.379±0.04 | 0.268±0.04 | 0.275±0.04 | 0.369±0.05 |
| ***gcvT*::Tn** | 0.469±0.05 | 0.452±0.07 | 0.511±0.06 | 0.482±0.04 | 0.098±0.03 | 0.262±0.04 | 0.42±0.04 |
| ***LP2387*::Tn** | 0.487±0.04 | 0.451±0.04 | 0.002±0.01 | 0.335±0.04 | 0.3±0.02 | 0.3±0.01 | 0.529±0.05 |
| ***dUTPase*::Tn** | 0.29±0.04 | 0.255±0.04 | 0.258±0.04 | 0.099±0.04 | 0.174±0.04 | 0.161±0.04 | 0.164±0.04 |
| ***bkdA1*::Tn** | 0.247±0.04 | 0.316±0.04 | 0.252±0.04 | 0.127±0.04 | 0.196±0.04 | 0.073±0.04 | 0.025±0.01 |
| ***pdxS*::Tn** | 0.169±0.04 | 0.166±0.04 | 0.146±0.04 | 0.052±0.04 | 0.103±0.04 | 0.096±0.04 | 0.142±0.04 |
| ***hom*::Tn** | 0.234±0.04 | 0.265±0.05 | 0.262±0.04 | 0.064±0.05 | 0.155±0.04 | 0.155±0.05 | 0.223±0.05 |
| ***pheA*::Tn** | 0.42±0.04 | 0.593±0.08 | 0.489±0.06 | 0.339±0.07 | 0.303±0.04 | 0.257±0.06 | 0.452±0.04 |
| ***0800*::Tn** | 0.237±0.05 | 0.177±0.05 | 0.185±0.04 | 0.06±0.01 | 0.002±0.04 | 0.132±0.04 | 0.205±0.05 |
